# A Study on Green Advertising Effectiveness in the Perspective of Image Proximity

**DOI:** 10.3389/fpsyg.2021.568189

**Published:** 2021-08-31

**Authors:** Guanghua Sheng, Qing Xia, Beibei Yue, Yuqi Li

**Affiliations:** ^1^School of Business, Jilin University, Changchun, China; ^2^AVIC Securities, Aviation Industry Corporation of China, Changchun, China

**Keywords:** image proximity, spatial distance, mental imagery, experience product, search product, advertising attitude, product attitude

## Abstract

Based on the construal level theory (CLT), this study discusses the effects of congruence between image proximity and product type on advertising attitude and product attitude from the perspective of spatial distance and investigates the mediating role of mental imagery. Data are collected using two laboratory experiments and one online experiment. A two-way ANOVA is used to test the interaction between image proximity and product type, and a bootstrap analysis is used to test the mediating role of mental imagery. The result shows that: (1) For search products, compared with full-length shots, the close-up shots of environmental information can enable consumers to generate more positive advertising attitude and product attitude. For experience products, the full-length shots of environmental information can enable consumers to generate more positive advertising attitude and product attitude than the close-up shots. (2) The congruence effect of image proximity and product type has an impact on advertising attitude and product attitude through mental imagery. This research uses different kinds of image proximity to express environmental information about green products and tries to interpret the effectiveness of green advertisements from a new perspective.

## Introduction

As corporate social responsibility increases on sustainable development, the green advertising strategy has become an important tool to enhance the green corporate image in green marketing (Dangelico and Vocalelli, 2017). Many studies tend to explore how to increase the persuasion of green advertising (Nyilasy et al., 2013; Chang, 2015). In the field of print advertisement, picture and text are well-known to be two essential tools for advertising to deliver product information effectively, and previous studies further indicate that the picture can be more impressive instead of the text (MacInnis and Price, 1987; Scott, 1994; Pieters and Wedel, 2004; Kergoat et al., 2017). Past studies on advertising pictures are mainly divided into two categories: realistic images and unrealistic images (Kim B. K. et al., 2019). Realistic images are defined as actual images of products which are consistent with the real item that people see, such as the suitable layout of visual elements, the key attributes, and the attractive color, etc. (Pieters and Wedel, 2004; Amit et al., 2009). Unrealistic images are defined as those seem similar to the real product images, but do not exist in the real world, for example, the organic food depiction by digital illustrations looks less attractive than the real organic food (Scott, 1994; Septianto et al., 2019). Although previous studies suggest that visual imagery is crucial to achieving effective advertising goal (Pieters and Wedel, 2004; Kergoat et al., 2017), limited studies have paid attention to visual imagery in green advertising.

The existing studies on the visual imagery of green advertising mostly focused on “what to express” (Chang, 2015; Septianto et al., 2019; Lim et al., 2020), that is, the content of visual imagery. For example, Septianto et al. (2019) argued that different advertising visuals (photographs vs. illustrations) may differ in conveying information; furthermore, compared to photographs, illustrations with altruistic appeals are able to increase the effectiveness of green advertisement. Meanwhile, Lim et al. (2020) demonstrated that the color of visual images influenced the advertising efficiency, and the results showed that the green visual images were more effective than gray visual images inactivating the pro-environmental awareness of an individual, thereby generating more positive attitude toward green advertising. Wang et al. (2020) found that the types of anthropomorphic images (people vs. animals) influence green advertising effectiveness differently. Aforementioned researches cannot address questions about the way to deliver content in visual imagery of green advertising. That is, how to highlight and deliver the green attributes information of products, such as healthy, energy-efficient, and eco-friendly, has not been fully investigated.

In general, advertising, to better attract the attention of the consumers on information of visual imagery, current studies mainly focus on the way of picture presentation, such as the picture order of the model and the product (Aydinoglu and Cian, 2014), the proper layout of visual elements (Cavallo and Piqueras-Fiszman, 2017), and visual metaphor (Delbaere et al., 2011), while neglecting the differences in the visual angle which is also a good way to influence picture presentation. Different visual angles of picture presentation will trigger different image elaboration process of the consumers and ultimately form different perception and preference of advertisements. Rooney and Balint (2018) investigated that close-up shots (vs. full-length shots) of sad expressions can attract more attention from individuals. Compared to a camera in the distance, a nearby camera will catch a smaller viewing area and more product details which make the consumers perceive the product as spatially close, thereby generating more positive advertising and brand attitude to rational appeals rather than emotional appeals (Kim K. et al., 2019). Further, Kim K. et al. (2019) proposed the concept of image proximity, which referred to different image distances (far vs. near) due to image angles and can be categorized as a full-length shot with larger viewing area and a close-up shot with more image details. However, the mechanism by which image proximity affects advertising attitude is still unanswered. In addition, research on image proximity has yet to be extended to green advertising. Consequently, this study will investigate the influence mechanism of image proximity on the reactions of the consumers to advertising, including advertising attitude and product attitude.

## Literature Review and Hypotheses Development

### Image Proximity

When mentioned about advertising pictures, the distinctive point of this paper should be the discussion over the spatial distance, namely image proximity. Image proximity refers to different image distances (far vs. near) due to image angles, which reflects the degree of image spatial distance perceived by consumers due to the visual angle, and can be categorized as a full-length shot and a close-up shot (Kim K. et al., 2019). The full-length shot with a longer spatial distance means a complete display of the whole product, while a close-up shot with a shorter spatial distance means a display of the concrete details of the product.

Construal level theory (CLT) offers insight into reasons why consumer response ranges from two types of image proximity. CLT has been used to explain decision-making and judgment of people by construing objects as psychologically distant or near. The theory assumed that the psychological distance (spatial, temporal, and social) between people and cognitive objects can affect the construal level (Fujita et al., 2006; Mi et al., 2020). That is, compared to low construal levels, high construal levels are viewed as more abstract, coherent, and decontextualized. When the psychological (spatial, temporal, and social) distance is far vs. near, the object can be construed as more abstract vs. concrete (Amit et al., 2009; Trope and Liberman, 2010). As psychological distance increases, people construe objects as more abstract, which are associated with high construal levels (Trope et al., 2007). Some studies have found that, compared to a short-distance location, people are more willing to believe that typical events will happen in a longer distance (Boroditsky, 2000; Henderson et al., 2006; Trope and Liberman, 2010). Bar-Anan et al. (2007) also argued that people construe the same persuasive information differently depending on the psychological distance. According to the theory, when people view the visual imagery of a full-length shot, they will describe the picture with more abstract words, which are associated with a high construal level. Conversely, when people view the visual imagery of a close-up shot, they will describe the picture with more specific and concrete words, which are associated with a low construal level.

In the field of advertising, CLT, as a theoretical support or research construct, has been widely used (Chang et al., 2015; Kim K. et al., 2019). Chang et al. (2015) argued that there was a congruence effect between framed messages (gain vs. loss) and construal levels (high vs. low), in which emphasizing the positive consequences of buying green products with a high construal level can generate more positive green advertising attitude. Kim K. et al. (2019) found that the effect of congruity between spatial distance (far vs. near) and advertising appeals (rational vs. emotional) was significant, and compared with the closer spatial distance, the far spatial distance was positively associated with emotional appeal rather than rational appeal. In this study, we thought different image proximity in green advertisements can evoke the emotional response of consumers through different paths and methods. When viewing product advertisements with full-length shots, consumers focus on the product as a whole, thereby perceiving the farther spatial distance. Under the circumstances, the abstract motive is playing a dominant role and a high construal level is activated to evoke a positive emotional response. When viewing product advertisements with close-up shots, consumers focus on the specific details of the product, thereby perceiving the closer spatial distance, in which the situations of the concrete motive plays a dominant role and starts with a low construal level to evoke a positive emotional response.

### The Congruence Effect Between Image Proximity and Product Type

It is extremely important to clarify the boundaries of product type for a marketing strategy of a company. Existing research has divided product types into various types, such as utilitarian and hedonic products, durable and non-durable products, and tangible and intangible products (Abreu et al., 2020; Zhou et al., 2021). According to information characteristics, the literature has identified two types of product type, search and experience products (Nelson, 1970). The distinction happens in the stage of evaluating information about quality, if consumers have the ability to evaluate information about quality in this period before purchasing products, which is called search product, resembling electronic products, computers, etc. (Mudambi and Schuff, 2010; Kim E. et al., 2019). For instance, they can search the function parameters, such as the processor, camera pixels, and running memory of smart phones in advance (Bei et al., 2004). Otherwise, the products are called experience products, which are evaluated after buying or using the products, such as milk, perfume, etc. (Mudambi and Schuff, 2010; Kim E. et al., 2019). For example, for the same organic milk, different consumers have different tastes (Bei et al., 2004). Attributes, such as health, safety, energy-saving, and low-carbon are key attribute features of green products, and are also the information that consumers focus on during the information search phase and product experience in the process of purchasing green products (Schmuck et al., 2018; Sun et al., 2018). Therefore, it becomes crucial for companies to better present the environmentally friendly information of different categories of green products in green advertising.

Previous studies found that the congruence effect can effectively improve the speed and accuracy of information dissemination, giving consumers intuitive feelings and then have a positive impact on the consumer attitude (Reber et al., 2004; Habitzreuter and Koenigstorfer, 2021). Academia has done much work on the congruence effect between advertising picture and product type. Existing research has investigated that there is a congruence effect between advertising visuals (photograph vs. illustrations) and food type (organic food vs. traditional food) because of the different information processing modes (Septianto et al., 2019). The organic food matches with the illustrations, starting with a high construal level, whereas the traditional food matches the photograph, starting with a low construal level, which can generate positive attitude toward advertising. Other researchers verified that there was also a matching effect between advertising message format and product type. For a durable product, the textual message will make consumers to possess higher purchase intention, while for nondurable products, the pictorial message is much better (Kim and Song, 2019). Further studies show that different picture types (person or product) can generate different attitudes for consumers with high (vs. low) self-esteem (Aydinoglu and Cian, 2014). Kim K. et al. (2019) discussed the congruence effect between advertising pictures with different spatial distance and advertising appeals, demonstrating that positive consumer response can be formed when emotional advertising appeals matches with farther spatial distance. Although the existing research has discussed the congruence effect of advertisement picture and product type from diverse angles (Kim K. et al., 2019), research on the congruence effect between advertisement picture and product type from the perspective of spatial distance is still insufficient.

As mentioned above, for the search product, its quality depends on the objective attributes; consumers can develop a certain understanding of the product attributes by searching for relevant product information in advance, then quantifying the relevant quality parameters of the product. For example, they can search the function parameters, such as the processor, camera pixels, and running memory of smart phones in advance (Bei et al., 2004). The information of search product is relatively specific, which can evoke the low construal level of the consumers. Under such condition of low construal level, people usually pay attention to more specific and standardized information from the perspective of minor details (Trope and Liberman, 2003, 2010). When green advertising picture of the product uses close-up shots, consumers will perceive the close spatial distance, starting the low construal level to process specific environmental information, such as carbon labels and packaging color in the green product. At the same time, the search product highlights more specific, partial, and unorganized environmental information, such as energy consumption signs on energy-efficient refrigerators which can be more easily expressed by product pictures with a closer space distance. Therefore, comparing to full-length shots with a higher abstract level, search products are more closely matched with close-up shots, and are more able to generate positive advertising attitude and product attitude.

For the experience product, its quality depends on the subjective attributes which can be found only after purchasing or using; different consumers have different perceptions about the same product quality so they cannot quantify their related quality parameters. For example, for the same organic milk, different consumers have different tastes (Bei et al., 2004). The information of experience product is relatively abstract which launches with a high construal level by consumers. In the case of high construal level, people usually focus on more abstract and non-standardized information from the main and core perspectives (Trope and Liberman, 2003, 2010). When the green advertising picture of the product uses full-length shots, consumers will perceive the far spatial distance, starting the high construal level to process the overall perception of green products and other environmental information. At the same time, as the experience product highlights more abstract, integral, and structured environmental information, for example, the health concept conveyed by organic milk, it is easier to be expressed by product pictures with a longer distance in space. Therefore, compared to the specific close-up shots, experience products are more closely matched with full-length shots, and are more able to generate advertising attitude and product attitude. Hence, it is hypothesized:

H1a: Consumers will have more positive advertising attitude in response to the close-up shots (vs. full-length shots) of environmental information for the search product.

H1b: Consumers will have more positive product attitude in response to the close-up shots (vs. full-length shots) of environmental information for the search product.

H1c: Consumers will have more positive advertising attitude in response to the full-length shots (vs. close-up shots) of environmental information for the experience product.

H1d: Consumers will have more positive product attitude in response to the full-length shots (vs. close-up shots) of environmental information for the experience product.

### The Mediating Role of Mental Imagery

The concept of mental imagery refers to the sensory information stored in working memory, such as sound, smell, taste, and touch (MacInnis and Price, 1987). Some studies revealed that many external stimuli will act on the sensory organs of the people and generate different sensory information through mental imagery (Bone and Ellen, 1992; Babin and Burns, 1997; Lien and Chen, 2013); so consumers are able to make the product visualization after touching or seeing it. Research of related scholars verified that three external stimulations, namely words, instructions to imagine and pictures can effectively evoke the mental imagery of consumers (Unnava and Burnkrant, 1991). Miller and Stoica (2004) found that the pictures of beach scene can evoke positive mental imagery. Research on tourism advertising pictures confirmed that the advertising pictures of tourist destinations (such as the majestic mountains and rivers) will enable consumers to combine their previous experiences to form a vacation experience, which can effectively evoke the mental imagery of the consumers regarding the tourist destination (Walters et al., 2007). There could be a conclusion that the external stimulation, especially visual images, is an important source of mental imagery. So pictures in green advertising can generate mental imagery, whether it is a full-length shot that can show the overall green product or a close-up shot that can show details of the product.

Mental imagery is generally measured in two dimensions: elaboration and quality. Elaboration refers to the amounts of images in working memory and quality refers to the degree of vividness, clarity, firmness, and profundity in imagery (Walters et al., 2007). For green products, there will be more vivid and clear imagery if visual pictures can provide a large number of vivid environmental information, especially the two dimensions of elaboration and quality. When the search product with high standardization matches with close-up shots, it can better express the information of the product due to the specific and standardized visual environmental information delivering by shots, so consumers will evoke greater elaboration and quality in mental imagery. When experience products with low standardization match with full-length shots, it can better express the information of the product owing to abstract and non-standardized visual environmental information delivered by shots, so consumers will evoke greater elaboration and quality in mental imagery. Therefore, when the image proximity matches the product type, consumers will evoke greater mental imagery.

In real life, people can determine the characteristics of products by the imagination because of similarities between mental imagery and realistic stimulus, so people could associate characteristics of apples, pineapples, and bananas with the color, fragrance, and taste. Similarly, sensory information stored in working memory can not only estimate the characteristics of the products but also make associations with different emotional responses (Yoo and Kim, 2014). Consumers are able to reflect on their personal preferences by mental imagery of the products. Previous studies have shown that mental imagery can affect the behavior and attitude of the consumers by stimulating their positive emotional responses (Bone and Ellen, 1992; Miller et al., 2000). The clearer you are in your thoughts, the higher level of elaboration and quality of mental imagery, the more purchase intention you will do (Yoo and Kim, 2014). Mental imagery activated by green advertising pictures will enable consumers to have a clear understanding of the safety, health, and environmentally friendly attributes of green products, then will further associate and judge the positive impact on consumers themselves and the environment, thus forming a positive advertising attitude and product attitude. Mental imagery plays a mediating role in multiple situations (Walters et al., 2007; Bambauer-Sachse and Gierl, 2009; Krishna et al., 2016; Lee and Shin, 2020). Walters et al. (2007) suggested that mental imagery played a mediating role between advertising pictures of tourist destinations and travel intentions. Bambauer-Sachse and Gierl (2009) also found that mental imagery was the mediating variable between nostalgic advertisements and advertisement attitude. Krishna et al. (2016) demonstrated that mental imagery also played a mediating role between advertising visuals and advertising attitude in sensory marketing. Lee and Shin (2020) argued that mental imagery played a mediating role in the relationship between apparel names and product attitude. Based on the above discussion, it is hypothesized:

H2a: Mental imagery plays a mediating role in the congruence effect between the image proximity and product type on advertising attitude.

H2b: Mental imagery plays a mediating role in the congruence effect between the image proximity and product type on product attitude.

**Figure 1:**
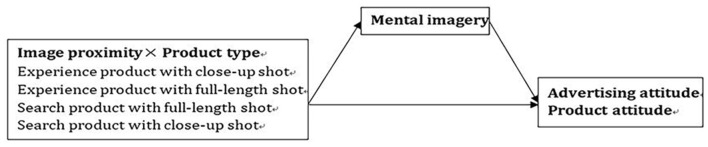
Research framework.

Based on the above, this paper proposed the congruence effect between image proximity and product type on product attitude and advertising attitude, and the mediating role of mental imagery in the above relationship. The research framework of this study is shown in Figure 1.

## Methods and Results

### Pretest

Prior to the main experiment, we conducted two pretests to determine the search and experience products used in the main experiment. Based on previous studies (Bei et al., 2004; Kim K. et al., 2019), eight green products that might be distinctively perceived as search or experience product were picked, namely energy-efficient refrigerator, energy-efficient air conditioners, solar water heaters, new energy vehicles, environmentally friendly laundry detergent, organic milk, bamboo pulp tissue, and environmentally friendly shampoos. Sixty MBA students (*M*_age_ = 32.62, *SD* = 5.93; 36 women) with purchase experience and skills participated in the first pretest recruited from a university in China. Participants were asked to score above eight kinds of green products, respectively, on a 7-point scale (1 = search product, 7 = experience product), to identify which product has more search or experience attributes. Participants perceived organic milk (*M* = 5.65, *SD* = 1.117) to be the experience product, followed by bamboo pulp tissue (*M* = 5.50, *SD* = 1.000), and new energy vehicles (*M* = 2.25, *SD* = 0.773) to be the search product, followed by energy-efficient refrigerator (*M* = 2.60, *SD* = 0.906). Thus, we selected organic milk as experience product and new energy vehicles as search product for the main experiment. Meanwhile, bamboo pulp tissue and energy- efficient refrigerator were selected as alternative experimental products.

To determine the specific brand of organic milk and new energy vehicles, we selected the top five organic milk brands and new energy vehicle brands with average monthly sales on the respective industry portals websites. Among them, the top five in the organic milk industry were Milk Deluxe, Satine, Organic, Bright Dairy, and Anchor, and the top five in the new energy vehicle industry are BYD, Tesla, Chery, KIA and BAIC Motor. Forty MBA students (*M*_age_ = 30.43, *SD* = 4.914; 20 women) recruited from a university in China participated in the second pretest. Participants were required to rate the brand familiarity of organic milk brands and new energy vehicle brands, on a 7-point scale ranging from 1 (very unfamiliar) to 7 (very familiar). Participants perceived Milk Deluxe (*M* = 6.33, *SD* = 0.829) and Tesla (*M* = 6.00, *SD* = 0.877), respectively, to be the most familiar brands. Thus, we selected Milk Deluxe as the brand of experience product and Tesla as the brand of search product.

### Experiment 1

Experiment 1 aimed to provide an initial investigation of H1, that is, the congruence effect between product type and image proximity. In advertising, the picture is one of the determinants of the evaluations and preference of consumers, and stimulates their subjective feelings and judgments (Aydinoglu and Cian, 2014). Thus, we used pictures as the stimulus material to reflect the image proximity and product type of advertising context.

#### Participants and Procedure

**Figure 2:**
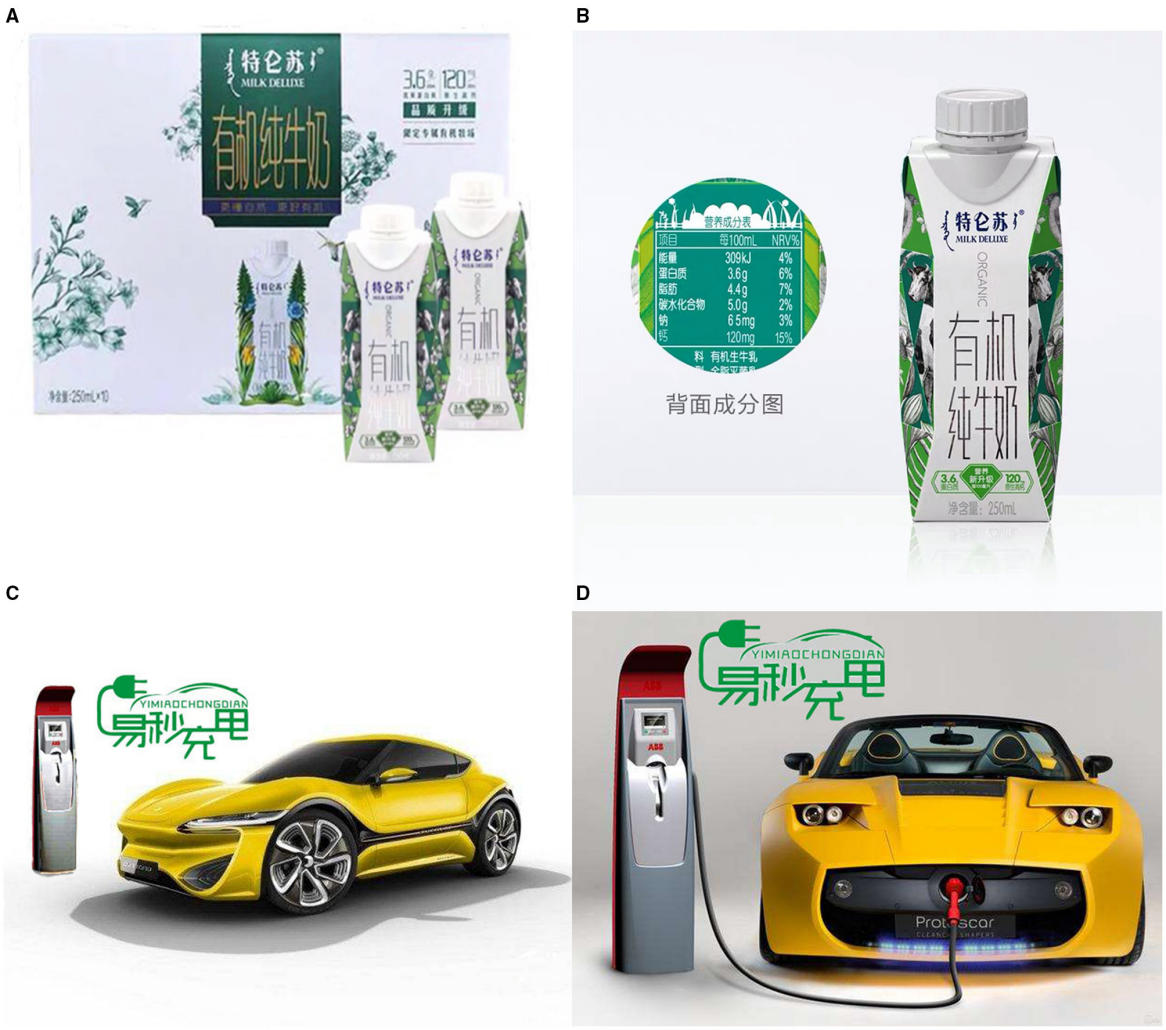
Organic milk used with full-length shot **(A)** and close-up shot **(B)**; New energy vehicles used with full-length shot **(C)** and close-up shot **(D)** (Image source: https://image.baidu.com).

To test the proposed hypotheses, a 2 × 2 (image proximity: full-length shot vs. close-up shot) (product type: search product vs. experience product) between-subjects design was used. According to a prior study on the influence of image proximity on advertising effectiveness (Kim K. et al., 2019), the number of subjects in each group was controlled at about 50–60. Thus, we chose a sample size of 50 participants per group and adjusted the number of subjects according to the size of the laboratory. A total of 220 MBA students from business schools with rich work and life experience in a university in China were invited to participate in this study, and 20 participants who did not complete the experiment as required were excluded. Eventually, 200 participants (*M*_age_ = 31.83, *SD* = 5.424; 124 women) completed the experiment. At the beginning of the experiment, participants were randomly assigned to view one of the four versions of advertising picture depicted different product types and image proximity. In experience product and full-length shot condition, the advertising picture showed a panoramic view of Milk Deluxe, depicting an overall packaging image of a crate of organic milk and two cartons of organic milk (Figure 2A). In experience product and close-up shot condition, the advertising picture showed a partial view of Milk Deluxe, depicting a packaging image of a carton of organic milk and nutrition ingredient list was posted on the left side of the packaging (Figure 2B). In search product and full-length shot condition, the advertising picture showed a Tesla car running on the road, and the charging pile conveyed environmentally friendly information about new energy vehicles on the left side of the picture (Figure 2C). In search product and close-up shot condition, the advertising picture showed a Tesla car was being charged, and the charging port connected to the charging pile highlighted environmentally friendly information on the left side of the picture (Figure 2D). All the product pictures used in the experiment materials, from free websites (https://image.baidu.com), have received permission.

After viewing the advertising picture, participants indicated their perceived spatial distance of the picture on a 7-point-Likert scale: 1 = very near to 7 = very far, to check whether the manipulation of image proximity in advertising pictures worked as intended. Then, participants were asked to fill out the 7-point-Likert scale of advertising attitude and product attitude, and complete personal information (e.g., age, gender). The advertising attitude (Cronbach's α = 0.882) was assessed by four items adapted from MacKenzie et al. (1986) and Kim K. et al. (2019): (1) I think the advertisement is interesting; (2) The advertisement is favorable; (3) I feel positive toward the advertisement; (4) I like the advertisement (1 = strongly disagree; 7 = strongly agree). The product attitude referred to the scale developed by Lee and Ang (2003): (1) The product is attractive; (2) I feel positive toward the product; (3) I react favorably to the product (1 = strongly disagree; 7 = strongly agree). Finally, each participant received a shopping coupon as a token of gratitude.

#### Results

##### Manipulation Checks

An independent sample *t*-test was conducted to examine whether respondents had different spatial distance of the close-up and full-length picture. In experience product condition, compared to close-up shot (*M* = 2.74, *SD* = 1.03, *n* = 47), full-length shot showed a higher mean spatial distance [*M* = 5.04, *SD* = 1.48, *n* = 50, *t*_(95)_ = 8.792, *p* < 0.001]. And again, in search product condition, compared to close-up shot (*M* = 2.96, *SD* = 1.13, *n* = 51), full-length shot showed a higher mean spatial distance [*M* = 5.02, *SD* = 1.44, *n* = 52, *t*_(101)_ = 8.077, *p* < 0.001]. The results confirmed that respondents perceived the close-up picture as near and the full-length shot picture as far.

##### Main Effect Analysis

A two-way ANOVA included image proximity (full-length shot vs. close-up shot) and product type (search product vs. experience product) as independent variables and advertising attitude and product attitude as a dependent variable, respectively. For advertising attitude, no main effects were identified for either image proximity [*F*_(1, 196)_ = 0.033, *p* = 0.857] or product type [*F*_(1, 196)_ = 0.345, *p* = 0.558]. Likewise, for product attitude, no main effects were identified for either image proximity [*F*_(1, 196)_ = 0.106, *p* = 0.745] or product type [*F*_(1, 196)_ = 0.226, *p* = 0.635]. But there existed significant interaction effects between image proximity and product type on advertising attitude [*F*_(1, 196)_ =13.731, *p* < 0.001] and product attitude [*F*_(1, 196)_ = 9.919, *p* < 0.01].

**Figure 3:**
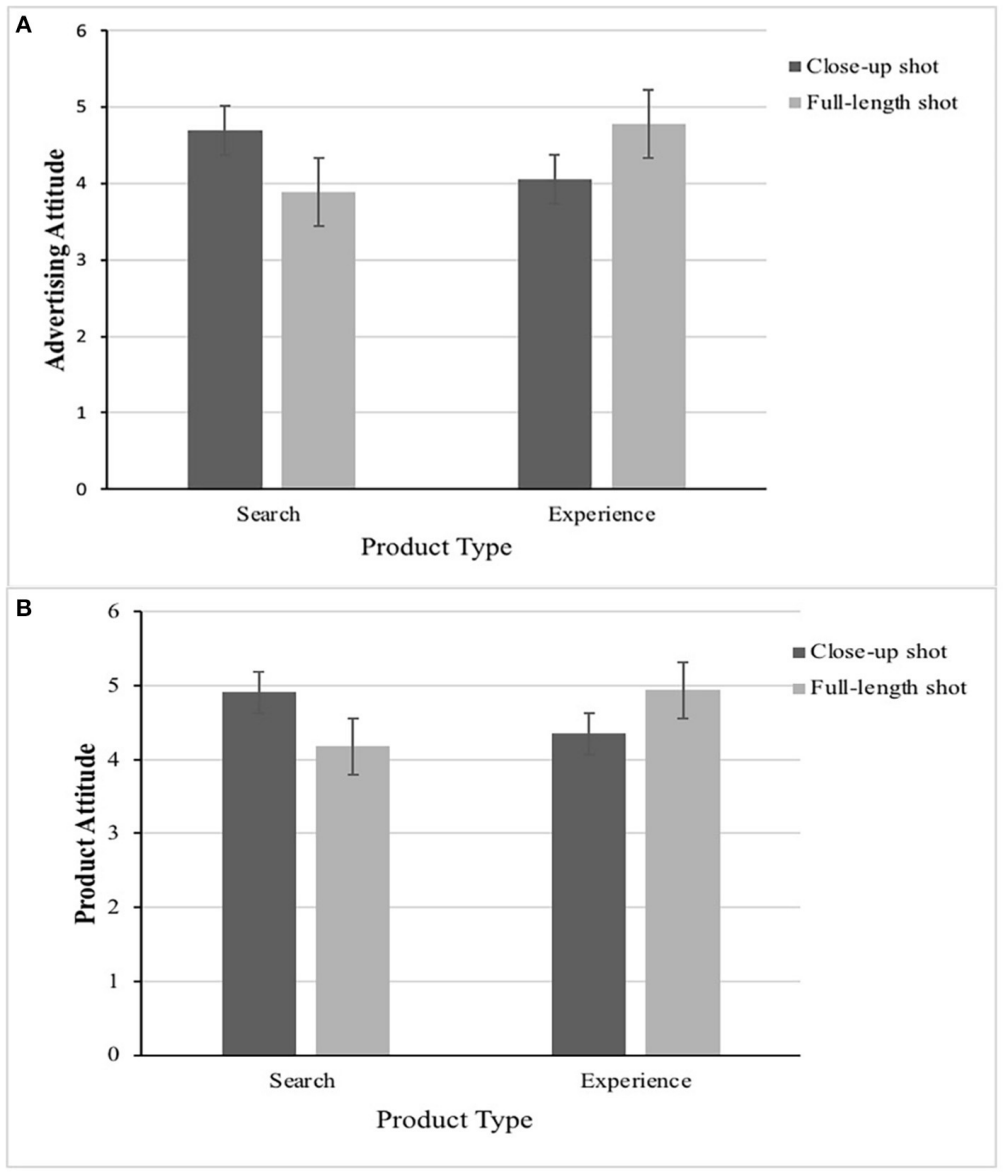
The congruence effect between image proximity (close-up vs. full-length) and product type (search vs. experience) on advertising attitude **(A)** and product attitude **(B)** in Experiment 1.

Particularly, participants who viewed search product expressed more positive attitude toward advertising in a close-up shot than a full-length shot (*M*_close−up_ =4.69 vs. *M*_full−length_ =3.89, *t*_(101)_ = 2.786, *p* < 0.01), in support of H1a. In contrast, participants who viewed experience product expressed more positive advertising attitude [*M*_close−up_ = 4.05 vs. *M*_full−length_ = 4.78, *t*_(95)_ =2.460, *p* < 0.05] in a full-length shot than a close-up shot, which supported H1c. When participants viewed search product, a close-up shot generated more favorable attitude toward the product than did a full-length shot [*M*_close−up_ = 4.91 vs. *M*_full−length_ = 4.18, *t*_(101)_ = 2.433, *p* < 0.05], in support of H1b. Contrary to the close-up shot, participants who viewed experience product expressed more positive product attitude [*M*_close−up_ = 4.35 vs. *M*_full−length_ = 4.94, *t*_(95)_ = 2.024, *p* < 0.05] in a full-length shot, which supported H1d. Thus, H1a, H1b, H1c, and H1d were supported. The result is as shown in Figures 3A,B.

#### Discussion

Hypothesis 1 predicted a congruence effect between product type and image proximity, such that participants who viewed search product would express more favorable attitude toward the close-up shot ad than the full-length shot ad, whereas participants who viewed experience product would express more favorable attitude toward the full-length shot advertisement than the close-up shot advertisement. The current study successfully verified this hypothesis using advertising picture of authentic brands. However, the internal mechanism, that the effects of congruity between image proximity and product type affect attitude toward advertising and product has not been explored. In addition, previous studies have shown that consumers may have certain established preferences for an authentic brand, that is, authentic brand may affect the response of consumers toward advertising pictures (Velasco Vizcaíno and Velasco, 2019). Thus, the next study was conducted to provide an explanation using advertising picture of fictitious brands.

### Experiment 2

Experiment 2 was conducted to explain how mental imagery can mediate the congruence effect between image proximity and product type on advertising and product attitude, and further investigate Hypothesis 1. As mentioned earlier, we utilized fictitious brands in Experiment 2 to eliminate the interference of brand preference. Furthermore, we replaced experimental products, which were able to examine the generalizability of the experimental results.

#### Participants and Procedure

**Figure 4:**
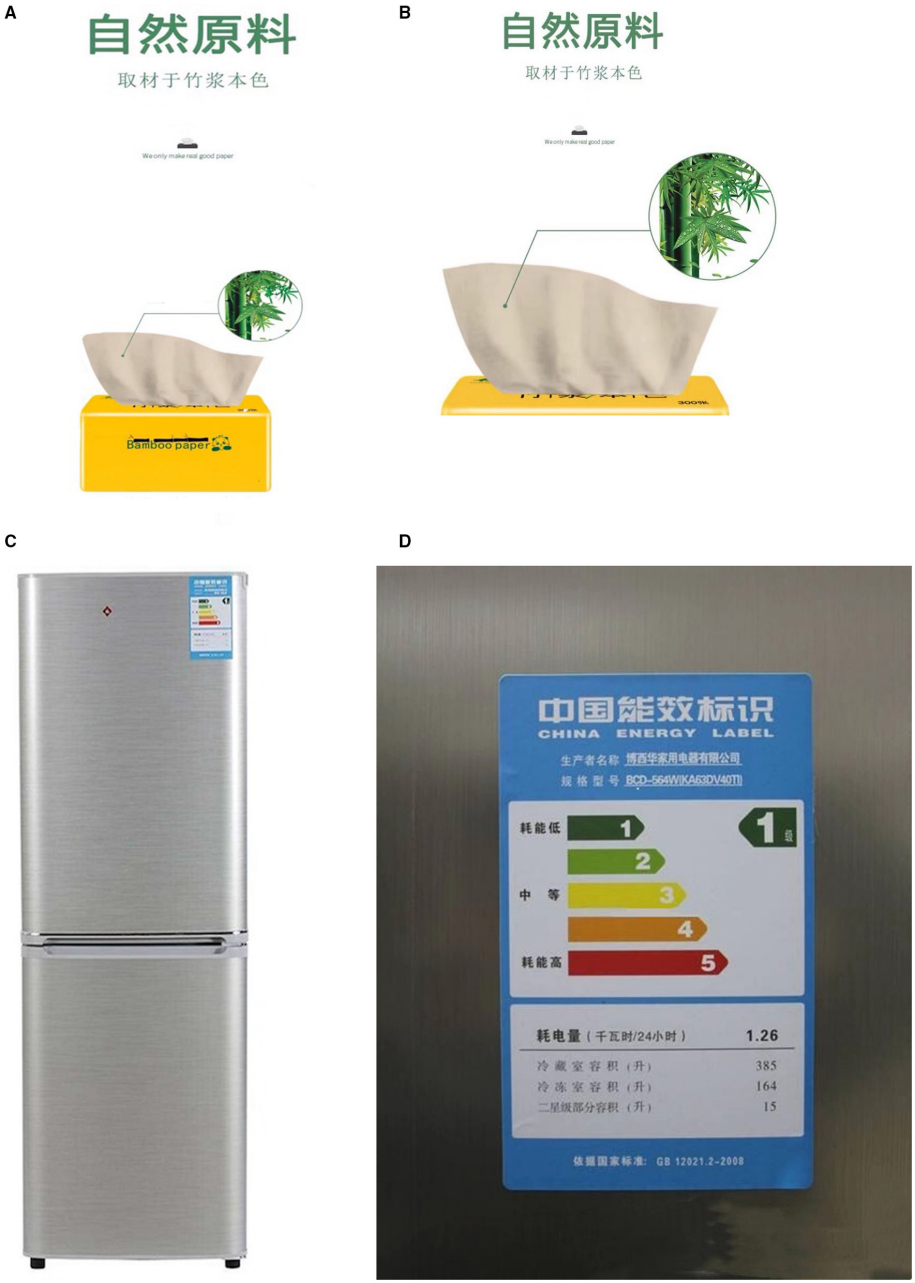
Bamboo pulp tissue used with full-length shot **(A)** and close-up shot **(B)**, Energy efficient refrigerator with full-length shot **(C)** and close-up shot **(D)** (Image source: https://image.baidu.com).

A total of 240 MBA students recruited from business schools of a university in China were randomly assigned to a 2 × 2 (image proximity: full-length shot vs. close-up shot) (product type: search product vs. experience product) between-subjects design. All participants were exposed to the similar experimental stimuli as in Study 1, except that we used different products and fictitious brands. Alternative products determined by the first pretest, bamboo pulp tissue, and energy-efficient refrigerators, respectively, were selected as experience product and search product for the main experiment. In experience product and full-length shot condition, the advertising picture showed a panoramic view of Kamihiki, a fictitious face tissue brand, and the image of bamboo as the raw material shown on the top right corner of the picture conveyed environmentally friendly information (Figure 4A). In experience product and close-up shot condition, the advertising picture showed a partial view of Kamihiki, a fictitious face tissue brand, and the image of bamboo as the raw material shown on the top right corner of the picture conveyed environmentally friendly information (see Figure 4B). In search product and full-length shot condition, the advertising picture showed the stereoscopic image of energy-efficient refrigerator of the fictitious brand Perto, and the best energy conservation certification was posted on the upper right corner of the refrigerator conveyed the environmentally friendly information about the product (Figure 4C). In search product and close-up shot condition, the advertising picture showed a partial image of energy-efficient refrigerator of the fictitious brand Perto, and the best energy conservation certification conveyed environmentally friendly information about the product was enlarged (see Figure 4D).

After viewing the advertising pictures, the participants were first asked to answer the perceived spatial distance on a 7-point-Likert scale and then to fill in the scale of advertising attitude (Cronbach's α = 0.866) and product attitude (Cronbach's α = 0.812). As in Experiment 1, all subjects reported the measurements of advertising attitude developed by MacKenzie et al. (1986) and Kim K. et al. (2019) and product attitude developed by Lee and Ang (2003). Subsequently, participants were asked to fill in the 7-point-Likert scale of mental imagery (Cronbach's α = 0.897). The mental imagery was measured by nine items adapted from Walters et al. (2007) and Yoo and Kim (2014), in which the first five items measured the elaborate processing level of mental imagery, and the last four items measured the quality level of mental imagery: (1) The mental images that came to mind formed a series of events in my mind in which I was a part of; (2) I could easily construct a story about myself and the featured product based on the mental images that came to mind; (3) Whilst reviewing the advertisement many images came to mind; (4)The images that came to mind acted as a source of information about the featured product; (5)The mental images that came to mind were very clear and specific; (6) Overall, the images that came to mind while we examined the advertisement were sharp; (7) Overall the images that came to mind while we examined the advertisement were intense; (8) Overall the images that came to mind while we examined the advertisement were clear; (9) Overall the images that came to mind while we examined the advertisement were vivid. Finally, they were asked to complete personal information (i.e., age and gender). Upon completion, each participant received a shopping coupon as a token of gratitude. After excluding the subjects without completing the experiment, a total of 216 valid samples (*M*_age_ = 32.24, *SD* = 4.683; 117 women) were used for data analysis.

#### Results

##### Manipulation Checks

An independent sample *t*-test was conducted to examine whether the respondents had different spatial distance of the close-up and full-length picture. In experience product condition, compared to close-up shot (*M* = 3.12, *SD* = 1.166, *n* = 52), full-length shot showed a higher mean spatial distance [*M* =4.77, *SD* = 1.262, *n* = 52, *t*_(102)_ = 4.085, *p* < 0.001]. And again, in search product condition, compared to close-up shot (*M* = 2.89, *SD* = 1.139, *n* = 56), full-length shot showed a higher mean spatial distance [*M* = 4.55, *SD* =1.264, *n* = 56, *t*_(110)_ = 7.305, *p* <0.001]. The results confirmed that respondents perceived the close-up picture as near and the full-length picture as far.

##### Main Effect Analysis

An analysis of variance (ANOVA) was conducted to test two-way interaction effects of image proximity and product type on advertising attitude and product attitude. The results indicated the single main effect of the image proximity and product type was not significant, that was, neither advertising attitude [*F*_(1, 212)image proximity_ = 0.610, *p* = 0.435; *F*_(1, 212)product type_ = 1.178, *p* = 0.279] nor product attitude [*F*_(1, 212)image proximity_ = 0.414, *p* = 0.520; *F*_(1, 212)product type_ = 0.761, *p* = 0.384] were significant; But image proximity and product type had significant interaction effects on advertising attitude [*F*_(1, 212)_ = 25.928, *p* < 0.001] and product attitude [*F*_(1, 212)_ = 20.305, *p* < 0.001].

**Figure 5:**
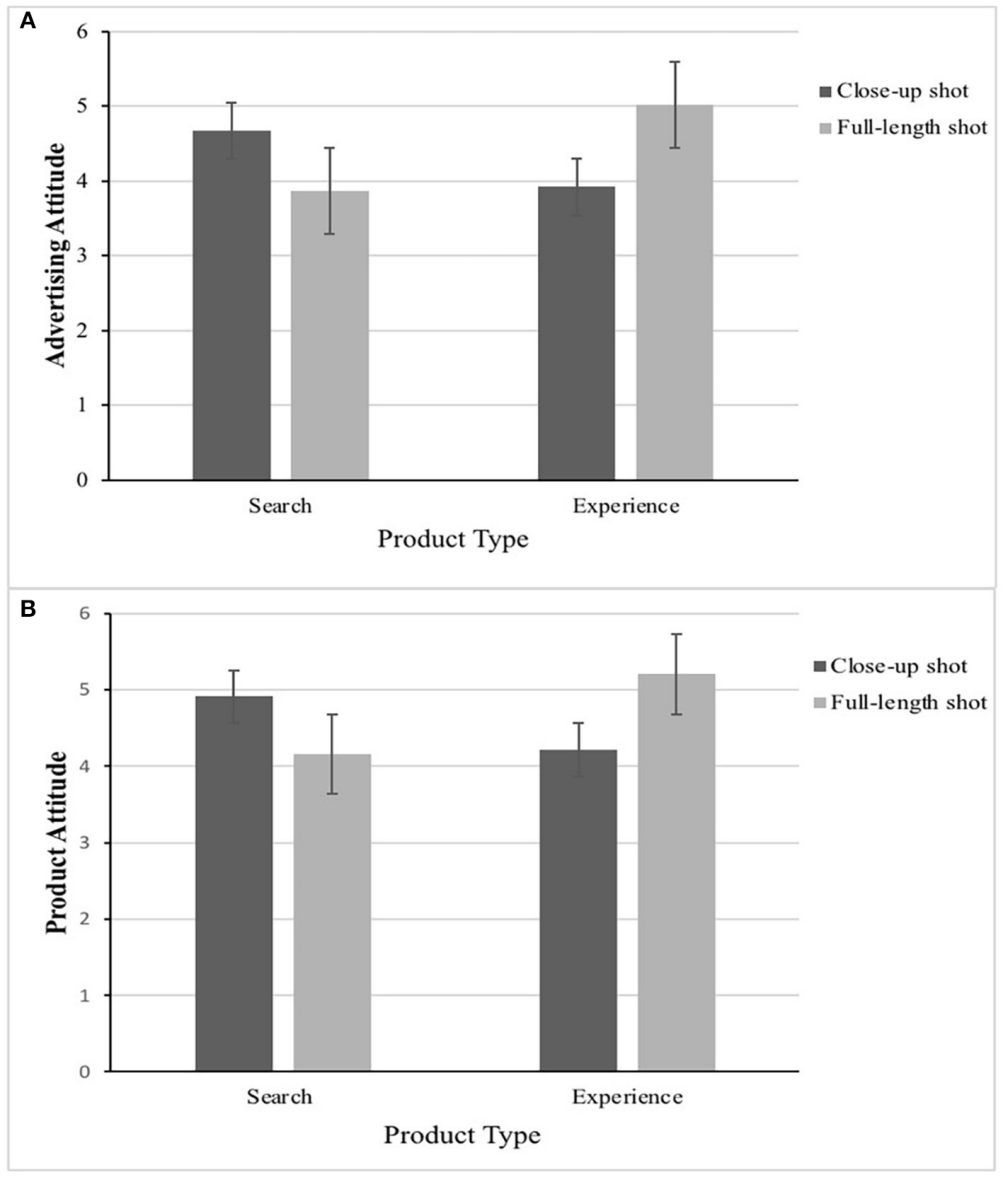
The congruence effect between image proximity (close-up vs. full-length) and product type (search vs. experience) on advertising attitude **(A)** and product attitude **(B)** in Experiment 2.

Particularly, participants who viewed search product expressed more positive advertising attitude [*M*_close−up_ = 4.67 vs. *M*_full−length_ =3.87, *t*_(110)_ = 3.101, *p* < 0.01] and product attitude [*M*_close−up_ = 4.91 vs. *M*_full−length_ = 4.16, *t*_(110)_ = 2.719, *p* < 0.01] in a close-up shot than a full-length shot, which supported H1a and H1b again. In contrast, participants who viewed experience product expressed more positive advertising attitude [*M*_close−up_ = 3.92 vs. *M*_full−length_ = 5.02, *t*_(102)_ = 4.085, *p* < 0.001] and product attitude [*M*_close−up_ = 4.21 vs. *M*_full−length_ =5.21, *t*_(102)_ = 3.672, *p* < 0.001] in a full-length shot than a close-up shot, which supported H1c and H1d again (as shown in Figures 5A,B).

##### Mediating Effect Analysis

To test whether the effects of congruity between image proximity and product type generated more favorable attitude toward the advertising and product by mental imagery, a mediation analysis was conducted using the PROCESS macro for SPSS (Model 4, *N*_bias−corrected bootstraps_ = 5,000; Hayes, 2013). Four purpose-designed experimental conditions were firstly coded as following: 1 = experience product with close-up shot; 2 = experience product with full-length shot; 3= search product with full-length shot; 4 = search product with close-up shot. In Model 4, we entered advertising attitude and product attitude, respectively, as the outcome variable (Y), four conditions (the interaction between image proximity and product type) as the independent variable (X), and mental imagery as the mediating variable (M). In the PROCESS macro of SPSS, experience product with close-up shot was set as the benchmark automatically (dummy coded as 0), experience product with full-length shot condition was dummy coded as 1 (D1), search product with full-length shot condition was dummy coded as 2 (D2), and search product with close-up shot condition was dummy coded as 3 (D3).

The summary of mediating analysis is depicted in Table 1. When advertising attitude was entered as outcome variable, the result showed that the mediating effect of mental imagery was significant (indirect effect _D1_ = 0.5499; indirect effect _D3_ = 0.4055) under experience product with full-length shot condition (D1) and search product with close-up shot condition (D3), with the confidence interval for both that did not include zero (95% CI_D1_ = 0.1683–0.9997; 95% CI_D3_ = 0.0698–0.8077), which supported H2a. Similarly, when product attitude was entered as outcome variable, the mediating effect of mental imagery was also significant (indirect effect_D1_ = 0.5476; indirect effect_D3_ = 0.4038) under experience product with full-length shot condition (D1) and search product with close-up shot condition (D3), with the confidence intervals for both did not contain zero (95% CI_D1_= 0.1550–0.9567; 95% CI_D3_= 0.0461–0.8145), which supported H2b. As expected, mental imagery played a mediating role in the two matching situations of experience product with full-length shot and search product with close-up shot.

**Table 1:** The mediation effect of mental imagery in Experiment 2.

	**IV**	**Effect**	**SE (boot)**	**LLCI**	**ULCI**
**DV:**	D1	0.5499	0.2092	0.1683	0.9997
Advertising attitude	D3	0.4055	0.1900	0.0698	0.8077
**DV:**	D1	0.5476	0.2063	0.1550	0.9567
Product attitude	D3	0.4038	0.1955	0.0461	0.8145

#### Discussion

Experiment 2 aimed to provide an explanation for the effects of congruity between image proximity and product type observed in Experiment 1. Consistent with the findings from Experiment 1, the results again suggested that differences between the close-up shot and the full-length shot may in fact depend on the product type (experience or search). Pictures with the close-up shot would fit better with search product, whereas pictures with the full-length shot would fit better with experience product. The result of the mediating effect further indicated that differences in attitude toward advertising and product might stem from the extent to mental imagery, and the extent to which varied across different image proximity and product type. Therefore, mental imagery played a mediating role, which supports H2. To further verify the robustness of the results, the next experiment changed the participants to verify all the proposed hypotheses.

### Experiment 3

The purpose of Experiment 3 was to further verify the robustness of the results. As in Experiment 2, we continued to utilize fictitious brands, but replaced the subject in Experiment 3.

#### Participants and Procedure

Participants were recruited from Credamo (https://www.credamo.com), a Chinese professional online survey platform, which has had 2 million respondents across China. We sent 220 individuals a link that included the experimental materials and the measurements of variables through this platform. The platform supported two-factor randomized trials, in which the participants were randomly assigned to one group and could not participate in the experiment repeatedly. Experimental stimuli of online Experiment 3 were exactly the same as Experiment 2, as shown in Figures 4A–D. Firstly, participants were randomly assigned to view one of the four versions of advertising picture depicting different product types and image proximity. And then, the participants were asked to report the perceived spatial distance, advertising attitude (Cronbach's α = 0.869), product attitude (Cronbach's α = 0.832), and mental imagery (Cronbach's α = 0.893) on a 7-point-Likert scale. As in Experiment 2, the advertising attitude was measured by three items adapted from MacKenzie et al. (1986) and Kim K. et al. (2019), and product attitude was measured by three items developed by Lee and Ang (2003), and the mental imagery was measured by nine items adapted from Walters et al. (2007) and Yoo and Kim (2014). After excluding the subjects who failed an attention check or complete the experiment with too long or too short time, 206 valid subjects (*M*_age_ = 35.29, *SD* = 9.918; 122 women) received monetary compensation for their participation.

#### Results

##### Manipulation Checks

An independent sample *t*-test was conducted to examine whether the respondents had different spatial distance of the close-up and full-length picture. In experience product condition, compared to close-up shot (*M* = 2.75, *SD* = 1.007, *n* = 52), full-length shot showed a higher mean spatial distance [*M* = 4.53, *SD* = 1.102, *n* = 51, *t*_(101)_ = 8.557, *p* < 0.001]. And again, in search product condition, compared to close-up shot (*M* = 2.96, *SD* = 1.148, *n* = 51), full-length shot showed a higher mean spatial distance [*M* = 4.85, *SD* =1.161, *n* = 52, *t*_(101)_ = 8.284, *p* < 0.001]. The results confirmed that respondents perceived the close-up picture as near and the full-length picture as far.

##### Main Effect Analysis

An ANOVA was conducted to test the two-way interaction effects of image proximity and product type on advertising attitude and product attitude. The results indicated the single main effect of the image proximity and product type was not significant, that was, neither advertising attitude [*F*_(1, 202)image proximity_ = 0.038, *p* = 0.846; *F*_(1, 202)product type_ =0.004, *p* = 0.947] nor product attitude [*F*_(1, 202)image proximity_ = 0.121, *p* = 0.728; *F*_(1, 202)product type_ = 0.083, *p* = 0.773] were significant; But image proximity and product type had significant interaction effects on the advertising attitude [*F*_(1, 202)_ = 16.903, *p* < 0.001] and product attitude [*F*_(1, 202)_ = 15.391, *p* < 0.001].

Particularly, participants who viewed search product expressed more positive advertising attitude [*M*_close−up_ = 4.51 vs. *M*_full−length_ = 3.62, *t*_(101)_ = 2.769, *p* < 0.01] and product attitude [*M*_close−up_ =4.70 vs. *M*_full−length_ = 3.93, *t*_(101)_ = 2.527, *p* < 0.05] in a close-up shot than a full-length shot, in support of H1a and H1b again. In contrast, participants who viewed experience product expressed more positive advertising attitude [*M*_close−up_ =3.60 vs. *M*_full−length_ = 4.57, *t*_(101)_ = 3.045, *p* < 0.01] and product attitude [*M*_close−up_ =3.92 vs. *M*_full−length_ = 4.84, *t*_(101)_ = 3.022, *p* < 0.001] in a full-length shot than a close-up shot, in support of H1c and H1d again.

##### Mediating Effect Analysis

As in Experiment 2, to test whether the effects of congruity between image proximity and product type generated more favorable attitude toward the advertising and product by mental imagery, a mediation analysis was conducted using the PROCESS macro for SPSS (Model 4, *N*_bias−corrected bootstraps_ = 5,000; Hayes, 2013). Four purpose-designed experimental conditions were firstly coded as following: 1 = experience product with close-up shot; 2 = experience product with full-length shot; 3= search product with full-length shot; 4= search product with close-up shot. In the Model 4, we entered advertising attitude and product attitude as the outcome variable (Y) respectively, four conditions (the interaction between image proximity and product type) as the independent variable (X), and mental imagery as the mediating variable (M). In the PROCESS macro of SPSS, experience product with close-up shot was set as the benchmark automatically (dummy coded as 0), experience product with full-length shot condition was dummy coded as 1 (D1), search product with full-length shot condition was dummy coded as 2 (D2), and search product with close-up shot condition was dummy coded as 3 (D3).

As shown in Table 2, when advertising attitude was entered as the outcome variable, the result showed that the mediating effect of mental imagery was significant (indirect effect_D1_ = 0.9742; indirect effect_D3_ = 0.8680) under experience product with full-length shot condition (D1) and search product with close-up shot condition (D3), with the confidence interval for both that did not include zero (95% CI_D1_= 0.3503–1.6311; 95% CI_D3_= 0.2017–1.4831), in support of H2a again. Similarly, when product attitude was entered as the outcome variable, the mediating effect of mental imagery was also significant (indirect effect_D1_ = 0.9072; indirect effect_D3_ = 0.8083) under experience product with full-length shot condition (D1) and search product with close-up shot condition (D3), with the confidence intervals for both did not contain zero (95% CI_D1_= 0.3183–1.4782; 95% CI_D3_= 0.2176–1.3919), in support of H2b again.

**Table 2:** The mediation effect of mental imagery in Experiment 3.

	**IV**	**Effect**	**SE (boot)**	**LLCI**	**ULCI**
**DV:**	D1	0.9742	0.3235	0.3503	1.6311
Advertising attitude	D3	0.8680	0.3238	0.2017	1.4831
**DV:**	D1	0.9072	0.2973	0.3183	1.4782
Product attitude	D3	0.8083	0.3022	0.2176	1.3919

#### Discussion

In Experiments 1 and 2, the subjects in the study mainly were MBA students, with life experience and skills to a certain extent. However, it is necessary to investigate whether the conclusions are general and applicable to the other consumer groups. Thus, in Experiment 3, the subjects were replaced with ordinary consumers with different educational backgrounds. Ultimately, Experiment 3 replicated the findings in Experiments 1 and 2 using different experimental materials and subjects, which enhanced the robustness of the results.

## Conclusion and Discussion

This study explored the influence mechanism of image proximity and product type on advertising attitude and product attitude through three experiments in green advertising context, and examined the mediating role of mental imagery. In the three experiments, the participants were randomly assigned to a 2 × 2 (image proximity: a full-length shot vs. a close-up shot) (product type: search product vs. experience product) between-subjects design. Experiment 1 demonstrated that the participants who viewed a search product (vs. experience product) expressed more positive advertising attitude and product attitude in a close-up shot (vs. a full-length shot). Experiment 2 demonstrated that the congruence effect of image proximity and product type enhances advertising attitude and product attitude through mediation of mental imagery. Experiment 3 replicated the findings in Experiments 1 and 2 by replacing different experimental materials and subjects. The contributions of this study to the existing theory can be divided into three parts. First, this study embeds spatial distance in green advertising picture context naturally, and demonstrates that an effective green advertising goal needs to apply different spatial distance in green advertising to present the environmental information. Previous study on green advertising pictures focused on the size, visualization, and presentation order of advertising pictures (Aydinoglu and Cian, 2014; Cavallo and Piqueras-Fiszman, 2017), mainly focusing on the characteristics of advertising pictures themselves (Rao et al., 2017), and rarely discussing from the perspective of the spatial distance of green advertising pictures. Limited study has shed some light on the impact of image proximity on advertising attitude and brand attitude (Kim K. et al., 2019), which has not been well validated and widely used in green advertisement, and it remains unclear how image proximity influences the attitude of the individuals. This study expands image proximity to the green advertisement that conveys environmentally friendly information, explores the impact of image proximity to consumer advertising attitude and product attitude, and enriched image proximity in the field of green consumption behavior. Second, this study demonstrates that mental imagery is the advanced cognitive process in green advertising persuasion mechanism which reveals how the image proximity and product type affect advertising attitude and product attitude. Previous study on the influence mechanism of green advertising basically revolved around simple cognitive variables, such as perceived value (Hyun et al., 2011). While past study has demonstrated that mental imagery played a vital mediation role in nostalgic advertisement and tourism advertisement (Walters et al., 2007; Bambauer-Sachse and Gierl, 2009), the mediating effect of mental imagery in green advertisement has not been fully discussed. Third, this study expands the application of CLT in green advertising context, which interprets the internal psychological mechanism between the spatial distance of the advertisement picture and attitude toward advertising and product. Despite prior researchers have applied CLT in the field of advertisement (Zhang et al., 2014; Chang et al., 2015; Septianto et al., 2019), most of which merely have focused on the explained influence mechanism of advertising appeal (“what to say”) on the persuasive effectiveness of advertising through the theory, and rarely applied the theory to understand “how to say” in green advertising, that is, how to express environmental friendly information.

In addition, this study offers two key implications for enterprises to develop advertising strategies in green marketing. One implication of this is the possibility that develops different advertising picture presentation strategies for different product types. When an enterprise pushes out a green product with search quality (or experience quality), it can use a close-up shot (or a full-length shot) in advertising marketing to express the specific (or abstract) environmentally friendly information, so as to activate the mental imagery of the consumers and thus improving their advertising and product attitudes. For example, displaying various performance parameters of new energy vehicle charging piles with a close-up shot or displaying natural pastures that produce organic milk with a full-length shot can enable consumers to understand the environmental characteristics of different products more clearly. Another implication of this is the possibility that utilizes advertising pictures as a visual expression way to develop a green advertising communication strategy. Since mental imagery depends on the amount and degree of the conscious images stored in the long-term memory, enterprises should not only focus on the amount of environmentally friendly information, but also highlight the vitality and attractiveness of visual image in the process of advertising design, to awaken the mental imagery of the consumers and strengthen their advertising and product attitude.

There are still some issues to further discuss. Firstly, the four conditions were compared with each other in the design of the experiment without a control group, resulting in the lack of a control condition which prevented using the control condition as the benchmark for the dummy coding. Secondly, this study explores the congruence effect of image proximity and product type (search products and experience products). Future studies can investigate whether this congruence effect can appear in other product types, such as utilitarian and hedonic products, high-involved products, and low-involved products. In addition, we can use field experiments to investigate consumer preferences for green advertising. Thirdly, future research can continue to expand the application of construal level theory in advertising strategy. In addition to considering the advertising pictures of different spatial distance, it can also explore the impact of social media of different social distance on the persuasive effectiveness of advertising.

## Data Availability Statement

The raw data supporting the conclusions of this article will be made available by the authors, without undue reservation.

## Ethics Statement

The studies involving human participants were reviewed and approved by Human Ethics Committees at Business School, Jilin University. The patients/participants provided their written informed consent to participate in this study.

## Author Contributions

GS involved in all steps of the study and provided critical revisions. GS, QX, and BY designed the study. QX and BY collected the data, analyzed the data, and write the manuscript. QX, BY, and YL revised the manuscript. All authors approved the final version of the article for submission.

## Conflict of Interest

YL was employed by company AVIC Securities, Aviation Industry Corporation of China. The remaining authors declare that the research was conducted in the absence of any commercial or financial relationships that could be construed as a potential conflict of interest.

## Publisher's Note

All claims expressed in this article are solely those of the authors and do not necessarily represent those of their affiliated organizations, or those of the publisher, the editors and the reviewers. Any product that may be evaluated in this article, or claim that may be made by its manufacturer, is not guaranteed or endorsed by the publisher.
